# Determinants of Proactive Low-Carbon Consumption Behaviors: Insights from Urban Residents in Eastern China

**DOI:** 10.3390/ijerph19106307

**Published:** 2022-05-23

**Authors:** Xiu Cheng, Jiameng Yang, Yumei Jiang, Wenbin Liu, Yang Zhang

**Affiliations:** 1 College of Economics and Management, Nanjing Forestry University, Nanjing 210037, China; yjm@njfu.edu.cn; 2 Zoucheng Bureau of Natural Resources and Planning, Jining 273500, China; jiangyumeilove@163.com; 3 School of Economics and Management, China University of Mining and Technology, Xuzhou 221116, China; 500107@cumt.edu.cn; 4 Department of Commerce, Jining Technician College, Jining 272100, China; jnzhangyang_2022@163.com

**Keywords:** proactive low-carbon consumption behavior, normative internalization, symbol, learning capacity, psychological empowerment

## Abstract

Proactive low-carbon consumption behaviors (*PLCBs*) are crucial to achieving carbon neutrality and identifying motivations for *PLCBs* is indispensable to changing individual consumption patterns. This study establishes a model by incorporating individual–group-level factors with psychological empowerment perception. The ordinary least-squares regression model was applied to identify the influencing factors of *PLCBs* with data collected from 1732 urban residents in eastern China. Results show that *PLCBs* are positively influenced by normative internalization, learning capacity, symbol concern, expertise level, and an environmentalism culture. In particular, the effect of learning capacity is the largest, with an influence coefficient of 0.271. A negative impact is observed between the consumerism culture and *PLCBs*. Moreover, psychological empowerment perception partly medicated the association between individual–group-level factors and *PLCBs*, and the maximum ratio of mediating effect to the full impact is 62.64%. The study sheds light on low-carbon-related behavioral management, and recommendations to promote *PLCBs* are further proposed.

## 1. Introduction

Greenhouse gas emissions caused by humans are considered the leading contributors to climate change [1]. According to the Intergovernmental Panel (IPCC) on Climate Change, the rise in global average surface temperature has a more than 95% chance to be caused by human activities [2]. Studies have revealed that more CO_2_ results from people’s daily consumption activities than from industrial production [3], constituting up to 50% [4] and 42% [5] of Japan’s and the United States’ total emissions, respectively. It was found that the CO_2_ from the consumption activities of one resident is 10, 12, and 14 tons annually in Tianjin, Beijing, and Shanghai, respectively [6]. The carbon emissions on the consumption side are estimated to be more when indirect carbon emissions are considered. It was found that 1.35 times direct as indirect carbon emissions are due to household consumption activities [7]. Furthermore, some scientists found that consumption-based indirect carbon emissions constitute about 80% of the total [8]. Consequently, reducing carbon emissions on the consumption side is vital to fulfill carbon neutrality and mitigate climate change.

Characterized by carbon reducing and energy saving, low-carbon consumption behaviors have captured global attention to change individual consumption patterns. Though much effort has been made, public response to behavioral consumption change is not encouraging. A two-year experiment indicated no measurable behavioral changes in UK residents’ energy consumption activities after information intervention [9]. Surveys have shown that more than half of consumers idle old electronic products instead of recycling them [10], and more than 90% of the respondents are absent from online clothing recycling activities [11]. In China, sales of new energy vehicles merely accounted for 2.69% of the total vehicle sales in 2016 [12], while the subsidies for one pure electric vehicle were as high as RMB 55,000 (approximately USD 8125 in 2016), which was about 30% of the average value of a new car at that time [13]. This dilemma is that individuals involuntarily practice low-carbon consumption behaviors, resulting in rebounds of opposing behaviors once the interventions (i.e., economic incentives and education) disappear. In contrast, proactive low-carbon consumption behaviors (*PLCBs*) are more conducive to changing consumption patterns and are more helpful in cultivating public habits of sustainable consumption behaviors. In this study, *PLCBs* are defined as consumption patterns in which consumers actively adopt low-emission and energy-saving behaviors in their daily consuming activities, including purchasing, using, and disposing. Unlike passive low-carbon consumption behaviors, *PLCBs* are created by individuals’ spontaneity and initiative rather than by external constraints [14], which highlights the motivations for enhancing social capital and personal reputation. Studies have shown that proactive pro-environmental behaviors are beneficial to overcoming free-riding activities [15] and building cooperation between the government and the public [16]. Therefore, *PLCBs* are pivotal to engaging the public in changing consumption patterns.

Large-scale studies have been conducted to identify the influencing factors for low-carbon consumption behaviors. Some situational factors, such as low-carbon technology [17], the price of low-carbon products [18], social norms [19], and infrastructure [20], are vital factors influencing low-carbon consumption behaviors. For example, Van der Linden [21] found that consumption of bottled water can be significantly reduced because of social norms and environmental information. Poortinga et al. [22] saw that situational factors would become more crucial when more effort is required to practice pro-environmental behaviors. Psychological factors also have effects on individual behaviors. It has been confirmed that attitude [23], environmental values [24], ecological emotions [25], and perceived behavior control [26] are key variables that influence individual low-carbon consumption behaviors. Cheng et al. [27] discovered that symbolic value positively affected personal pro-environmental behavior choices. Taking Portugal and Brazil as examples, Bertooldo and Castro [28] found that class identification was more effective than social norms when forecasting organic food purchase behavior. Demographic factors (i.e., gender, age, family size, and educational level) have impacted individual behavioral choices [29]. In general, the present studies mainly focus on individual-level factors, with group-level characteristics neglected. In particular, integrative research from an individual–group perspective has not been adequately conducted. Furthermore, prior studies have broadly failed some important motivations, such as normative internalization, learning capacity, symbol concern, and psychological empowerment perception.

The objective of this study is to identify influencing factors for *PLCBs* and propose targeted measures to promote *PLCBs*. Specifically, this study is devoted to uncovering the driving factors of *PLCBs* at the individual–group level, and the mediating effects of psychological empowerment perception between the associations of driving factors and *PLCBs*. The contributions of this study are as follows: (1) *PLCBs* are proposed and built as a composite of *PLCBs* for habit, *PLCBs* for decision, *PLCBs* for relationship, and *PLCBs* for pioneer. This fills the gap in proactive behavior for low carbon and provides a new perspective for behavioral intervention in the private sphere. (2) Motivations for *PLCBs* are proposed from an individual–group level, thus providing new perspectives and variables (i.e., normative internalization, learning capacity, and symbol concern) for future studies on individual pro-environmental behaviors. (3) The mediating effect of psychological empowerment perception is found in the association between individual–group-level factors and *PLCBs*, which deepens the understanding of generating mechanisms of low-carbon behavior in applied energy.

## 2. Theoretical Background and Hypotheses

### 2.1. Definition of Influencing Factors

Grounded theory was applied to identify the influencing factors of *PLCBs*, which included three steps. First, in-depth interviews of representative urban residents in eastern China were conducted to get first-hand information about their attitudes and cognition of *PLCBs*. Key factors affecting *PLCBs* should be excavated to find the influencing mechanism of *PLCBs*. Second, open, axial, and selective coding of the original materials was conducted to determine the influencing factors and paths. Finally, a theoretical saturation test was undertaken to build the theoretical model of *PLCBs*. Considering the forward-looking nature of *PLCBs*, it is required that the interviewees are aware of low-carbon consumption and have basic ideas about *PLCBs*. Participatory dialogues were elaborately established to guide the interviewees to start from their daily activities and focus on the interview content. The interview questions were focused on *PLCBs*, such as “Are you willing to practice *PLCBs* in daily life?” and “What factors will affect your practice of *PLCBs*?” Based on the analysis above, two categories of influencing factors were summarized. The individual-level factors include normative internalization, learning capacity, symbol concern, and psychological empowerment perception. The group-level characteristics are presented by expertise level, consumerism culture, and environmentalism culture. The definitions of the influencing factors are as follows:

- Normative internalization: the extent to which individuals incorporate reducing carbon emissions into their codes of conduct.
- Learning capacity: the ability to learn knowledge about low-carbon consumption.
- Symbol concern: the degree to which individuals are concerned about symbols extracted from low-carbon consumption behaviors.
- Psychological empowerment perception: individuals’ subjective judgment or psychological perception of management measures, opportunities, resources, and support required to complete specific work or action.
- Expertise level: the degree of authority people around individuals in their cognition and insight about low-carbon consumption behaviors.
- Consumerism culture: the social patterns in which individuals are encouraged to consume goods and services without constraints.
- Environmentalism culture: the social patterns in which individuals are encouraged to practice low-carbon behaviors.

### 2.2. Research Hypotheses

According to the theory of planned behavior, subjective norms will affect behavioral intention and influence an individual’s decision on pro-environmental behavior [23]. In value–belief–norm theory, the norm is presented by eco-moral awareness and is an essential factor influencing environmental actions in the private sphere [30]. Triandis [31] proposed the theory of interpersonal behavior and found a causal chain, namely “norms–social factors–behavior intention–behavior”. Normative internalization is the degree to which individuals incorporate protecting the environment into their codes of conduct. Individuals with a high level of normative internalization take the mainstream social norms as the constraints of behavioral choice rather than goals to be achieved. Therefore, normative internalization is supposed to motivate individuals to engage in low-carbon consumption behavior. Prior studies have confirmed the positive effects of normative internalization on pro-environmental behaviors. Using data collected from 4872 Australian respondents, Dean et al. [32] found that a more substantial level of social norms is conducive to maintaining water-saving behaviors. With two yearlong field experiments conducted, Anderson et al. [33] discovered that the probability of changing energy-using behaviors of individuals with a deep concern for the social norm is about three times that of low-level social norm. Consequently, residents with high normative internalization are more likely to perform *PLCBs*. Based on the discussions above, the following hypothesis was built:

**Hypothesis** **1** **(H1).** *Normative internalization positively affects PLCBs.*

Learning capacity presents individuals’ ability to update and broaden their knowledge about low-carbon consumption behaviors based on existing experience and expertise [34]. OECD [35] stated that learning capacity is crucial to managing conflicts and guiding individuals to be involved in organizational citizenship behavior. To cognitive learning theory, active learners can constantly update their cognition of pro-environmental behaviors via psychological structure adjustment and information processing [36]. According to social learning theory, individuals’ attitudes toward public social products can be shaped by conducting self-reflection on behavior clues [37]. It can be concluded that learning capacity influences individuals’ ability to process information and data at the internal level and impacts their compliance with social norms and group pressure from the external environmental level. Considering the information explosion, learning capacity has a more critical role in guiding decision makers to collect and analyze information obtained from the outside. Therefore, individuals with a higher learning capacity are more capable of correcting their biases and managing behavior conflicts; thus, they are more likely to adopt low-carbon consumption behaviors. Previous studies have confirmed the positive effect of learning capacity. It was found that knowledge sharing is helpful in reducing repetitive errors [38], and the public response to pro-environmental behaviors can be enhanced through learning and training [39]. Hence, the following hypothesis was proposed:

**Hypothesis** **2** **(H2).** *Learning capacity positively affects PLCBs.*

Symbol concern is the extent to which individuals pay attention to the added values extracted from low-carbon consumption behaviors. In line with role theory, both differences and similarities are pursued when individuals make comparisons with others [40]. When making a behavioral choice, some special symbols, such as personality, status, authority, and emotion, are endowed with low-carbon consumption behaviors, influencing individual behaviors. For example, the signs of “health”, “social responsibility”, and “environmentally friendly” can be conveyed to others when individuals adopt low-carbon behaviors. By the connection theory, the symbols can act to bind untied individuals and the specific behaviors. Moreover, individuals are more satisfied with the symbols when the association is high, which in turn enhances individuals to adopt the expected behavioral pattern [41]. Furthermore, symbol concern is beneficial to achieving self-esteem and self-coordination by activating individuals’ memory structures and information storage, thereby encouraging decision makers to practice low-carbon consumption behaviors [42]. The positive effects of symbol concern get support from past studies. It was confirmed that the symbolic attribute of new energy vehicles (i.e., opening, freedom, and environmentally friendly) is conductive to enhancing consumers’ purchase intentions [43]. Mondou et al. [44] discovered that the symbols embedded in the United States’ biofuel policies were multidimensional, tenacious, and affective predictors of policy-expected effects. Based on the above discussions, the following hypothesis was built:

**Hypothesis** **3** **(H3).** *Symbol concern positively affects PLCBs.*

Expertise level describes people around residents’ authority in their cognition and insight about low-carbon consumption behaviors. A high level of knowledge indicates that a lot of professional information and knowledge (i.e., skills, experience, and explanations for policy) can be provided for residents when faced with a behavioral choice. According to the theory of responsible environmental behavior, behavioral knowledge and environmental knowledge are the critical predictors of environmental behavior, which are the decisive factors for individuals to implement pro-environmental behavior [45]. Prior studies indicated that individuals behave environmentally when surrounded by professionals, which is in line with the theory of responsible environmental behavior. Using samples from Spain, Peña-Vinces et al. [46] found that others’ ecological knowledge and previous experience can increase consumers’ willingness to practice responsible consumption by purchasing or renting second-hand perinatal and infant clothes. Glick et al. [47] found that knowledge related to policy is the most critical predictor of water recycling behavior based on a sample of 1000 Americans. Andrei et al. [48] discovered three forms of knowledge (technical form of knowledge, process knowledge, and leadership knowledge). They stated that sharing these types of knowledge is the power to maximize the potential for energy management. Thus, it can be concluded that a level of expertise is helpful in promoting *PLCBs*, and thereby the following hypothesis was made:

**Hypothesis** **4** **(H4).** *Expertise level positively affects PLCBs.*

Consumerism culture refers to the social pattern in which individuals are encouraged to consume goods and services without constraints. In contrast, environmentalism culture is the social pattern in which individuals are encouraged to practice pro-environmental behaviors. The theory of interpersonal behavior indicates that norms affect behavior intentions and behaviors [31]. According to the theory of planned behavior, individual behaviors and their behavior intentions are predicted by their subjective norms [23]. In general, consumerism and environmentalism cultures are different social norms, representing the social pressure individuals perceive over whether they should take a particular action. The qualitative analyses indicated that respondents tend to endow specific symbols (i.e., status and wealth) to luxury goods and expensive services when influenced by the consumerism culture; residents are willing to practice low-carbon consumption behaviors when impacted by environmentalism culture. Previous studies have confirmed the different effects of the above two factors. Gu et al. [49] found that materialism is related to the decrease in pro-environmental attitudes and behaviors, and more energy is consumed when more materialistic regions are. It was found that environmentalism is positively associated with individual pro-environmental behaviors [50] and directly and indirectly affect adolescents’ actions by intergenerational transmission [51]. Consequently, it can be supposed that consumerism culture negatively influences *PLCBs*, while environmentalism culture can enhance *PLCBs*.

**Hypothesis** **5** **(H5).** *Consumerism culture negatively affects PLCBs.*

**Hypothesis** **6** **(H6).** *Environmentalism culture positively affects PLCBs.*

Psychological empowerment perception is an individual’s subjective judgment or psychological perception of the management measures, opportunities, resources, and support required to complete specific work or action [52], which can be divided into competence, meaning, self-determination, and impact. “Competence” demonstrates the perception of the ability of individuals to achieve the goal; “meaning” presents the value of a work or action evaluated by individuals; “self-determination” refers to individuals’ cognition of the level to which they can control the work; and “impact” shows the influence perception by individuals when taking work or action [53]. The in-depth interviews indicated that respondents’ meaning, autonomy, and efficacy could enhance their willingness to practice low-carbon consumption behaviors. Three possible explanations account for this. First, there are positive relationships between psychological empowerment perception and *PLCBs*. According to decision theory, the interest in a specific action has a decisive impact on the decision maker’s behavioral choice [54]. Individuals’ cognition of the meaning and implications involved in psychological empowerment is the quantification of this interest, which can inhibit or promote the action. In addition, “control” and “self-determination” represent the emotional experience of taking action, which are essential motivations for expected behavior. Hence, psychological empowerment perception would generate positive effects on *PLCBs*. Second, individual–group-level factors are associated with psychological empowerment perception. Studies have shown that individual–group-related factors have effects on psychological empowerment perception. For example, it was found that the impact of regulatory focus on waste-recycling behavior was mediated by psychological empowerment [55]; contextual antecedent (i.e., leadership and socio-political support) are strongly associated with psychological empowerment [56]. Finally, according to the analyses above, individual–group-level factors impact *PLCBs*. Therefore, psychological empowerment perception is assumed to mediate the association between individual–group-level factors and *PLCBs*, and the following hypothesis is made:

**Hypothesis** **7** **(H7).** *Psychological empowerment perception mediates the association between individual–group-level factors and PLCBs.*

**Figure 1:**
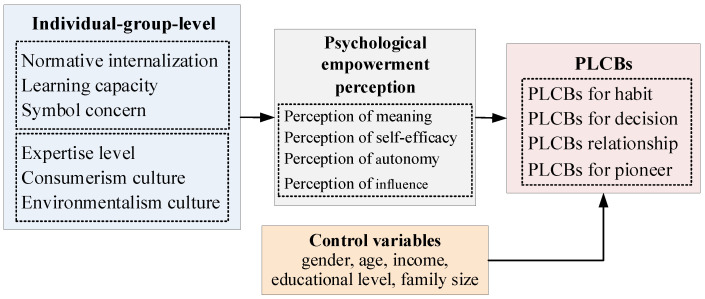
Theoretical model.

According to the hypothesis proposed above, the theoretical model built in this study is available in Figure 1.

## 3. Materials and Methods

### 3.1. Sample and Data Collection

**Figure 2:**
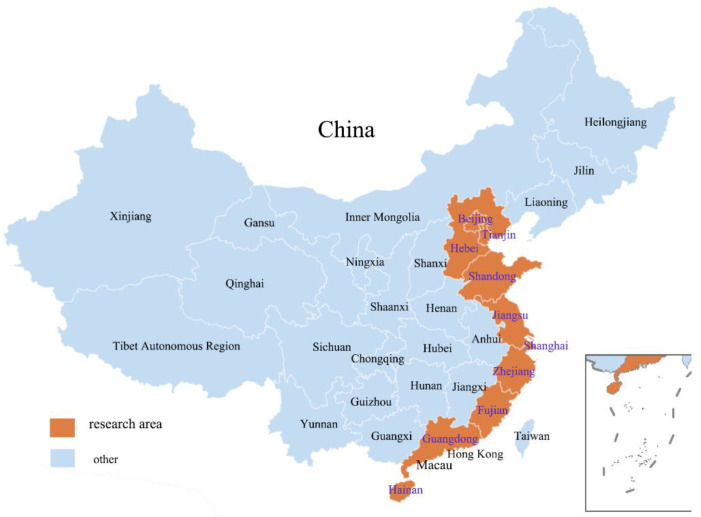
Research area.

This study selected eastern China as the research area, including Beijing, Tianjin, Hebei, Shanghai, Jiangsu, Zhejiang, Fujian, Shandong, Guangdong, and Hainan (Figure 2). There are three reasons for this. First, eastern China has captured researchers’ wide attention because of its severe environmental problems [57]. Second, high-paid and well-educated residents in the east of China desire a better living environment and are more willing to change their behaviors [58]. Third, as the first region to promote pro-environmental behavior in China, eastern China is equipped with various guiding policies, and residents are well-informed about low-carbon consumption [59].

With Nanjing selected as the research area, a preliminary investigation was implemented to test the reliability and validity of initial scales from 9 April 2021 to 28 April 2021. Questionnaires were issued through an online survey platform named “Wenjuanxing” (https://www.wjx.cn/, accessed on 1 May 2022), with 462 valid questionnaires collected. SPSS 22.0 was applied to conduct the test, and the results showed that good reliability and validity could be guaranteed for the initial scales.

From 25 May 2021 to 6 September 2021, the formal investigation was conducted via online and paper questionnaires. A stratified sampling method was used to determine the interviewees to make the demographic distributions of respondents reasonable and representative. Using the snowball sampling method, the URL of the online questionnaire was sent out to engage respondents via social media (i.e., WeChat 8.0.22, Tencent, Shenzhen, China). Moreover, we entrusted our friends and classmates in eastern China to distribute the online questionnaire. Paper questionnaires were issued in subway stations, food markets, shops, school gates, and parks to include residents not involved in the online survey. A one-to-one method, in which interviewers completed questionnaires according to respondents’ answers, was used for some older respondents. Before filling out the questionnaires, respondents were precisely informed of the critical variables involved in this study, such as *PLCBs*, symbol concern, normative internalization, and expertise level. Furthermore, all respondents were told that the information collected would be used only for academic research. A filtering process was applied to delete invalid questionnaires with contradictory and incomplete answers. Finally, a total of 1732 valid questionnaires were obtained after removing 523 invalid ones, with an effective recovery rate of 76.81%.

### 3.2. Measures and Scale Test

A five-point Likert scale ranging from 1 (“strongly disagree”) to 5 (“strongly agree”) was applied to measure *PLCBs*, normative internalization (*NI*), learning capacity (*LC*), symbol concern (*SC*), expertise level (*EL*), the consumerism culture (*CC*), environmentalism culture (*EC*), and psychological empowerment perception (*PEP*). According to Chen et al. [60], *PLCBs* are measured from habit, decision, relationship, and pioneer. Twelve items are built to measure *PLCBs*, and one example is “I am used to turning off the lights when leaving the room”. The scale of normative internalization is self-developed, and one example is “Saving water and electricity is what I should do well”. Adapted from Cheng et al. [61], three items were developed to evaluate learning capacity: “I refer to past experience when buying home appliances”. Consistent with Mandler et al. [62], four items are designed to measure symbol concern, and one example is “I can express my personality by practicing low-carbon consumption behaviors”. Three items evaluate the expertise level; one example is “People around me know a lot about the low-carbon consumption guiding policy”. There are three measurement items for consumerism culture, such as, for example, “It is widespread that people consume goods and services in large quantities”. Three items assess environmentalism culture, and one example is “Many people pay attention to emissions reduction and energy saving”. According to Thomas and Velthouse [53], 12 items are developed to measure psychological empowerment perception. One example is “I think it makes sense to practice low-carbon consumption behaviors”.

The reliability of the scales was tested by Cronbach’s α and composite reliability (CR). The Cronbach’s α value for each construct was greater than 0.7, and the CR was above 0.7 for all constructs involved in this study. Furthermore, the average variance extracted (AVE) values for each construct were higher than 0.5, indicating that the scale was reliable.

The validity was checked by confirmatory factor analysis. For each construct, the Kaiser–Meyer–Olkin was greater than 0.7, suggesting that the scale is suitable for factor analysis. The χ2/df, normed fit index, good fit index, comparative fit index, and root mean square error of approximation were 2.336, 0.942, 0.933, 0.916, and 0.049, respectively, suggesting that the measurement model is valid. All the items developed to measure construct were equipped with a standardized factor loading higher than 0.7 and significantly positive. Moreover, no double loading was observed in all constructs. Consequently, the scale has good validity. The measurement item of each variable and the reliability and validity analysis is available in Supplementary Materials.

### 3.3. Data Analysis Method

#### 3.3.1. Main Effects Test

According to the hypothesis proposed above, normative internalization, learning capacity, symbol concern, expertise level, consumerism culture, and environmentalism culture are independent variables, psychological empowerment perception is mediating variable, and *PLCBs* are dependent variables in this study. The ordinary least-squares evaluating model was applied to examine the main effects between independent variables and dependent variables, as shown in Equation (1):

(1) $$PLCBs_{i}=\alpha_{10}+\alpha_{11}NI_{i}+\alpha_{12}LC_{i}+\alpha_{13}SC_{i}+\alpha_{14}EL_{i}+\alpha_{15}CC_{i}+\alpha_{16}EC_{i}+\alpha_{17}{\sum Control}_{i}+e_{1i}$$

where *i* denotes the *i*th respondent, *NI*, *LC*, *SC*, *EL*, *CC*, and *EC* represent normative internalization, learning capacity, symbol concern, expertise level, consumerism culture, and environmentalism culture, respectively. “*Control*” denotes control variables, including age, gender, income, educational level, and family size.

#### 3.3.2. Mediating Effect Test

Three steps are carried out to test the mediating effect of psychological empowerment perception. In step 1, the coefficients (*c*_1_) of the individual–group-level factors in the regression model of individual–group-level variables and *PLCBs* are determined. If *c*_1_ is not significant, there are no mediating effects. In step 2, the coefficients (*a*) of the individual–group-level factors in the regression model of individual–group-level variables and psychological empowerment perception are obtained. In step 3, the coefficients (*c*_2_) of the individual–group-level factors, (*b*) psychological empowerment perception in the regression model of individual–group-level variables, psychological empowerment perception, and *PLCBs* are obtained. There are no mediating effects if neither a nor *b* is significant; there are full mediating effects if *a* and *b* are significant, and *c*_2_ is not significant; there are partly mediating effects if *a*, *b*, and *c*_2_ are significant, and the ratio of the mediating effect to the full impact can be calculated as follows:

(2) $$RME=a\times b/c_{1}\times 100\%$$

where *RME* refers to the ratio of the mediating effect to the full impact.

## 4. Results

### 4.1. Descriptive Statistics

The descriptive statistics of the variables involved in this study are available in Table 1. The mean value of *PLCBs* is 3.51, suggesting that residents are positively responsive to *PLCBs*. The mean value of *NI* is less than 3, which implies that the normative internalization of residents is not encouraging. The mean value and standard deviation of *CC* are 3.75 and 1.137, respectively, denoting that consumerism is common and a big difference in individual perception of the culture of consumerism. The great mean value of *SC* indicated that respondents had paid attention to the symbols embedded in *PLCBs*. The mean value of *EL* is 3.04, representing a lack of professionals with low-carbon-related knowledge around respondents.

**Figure 3:**
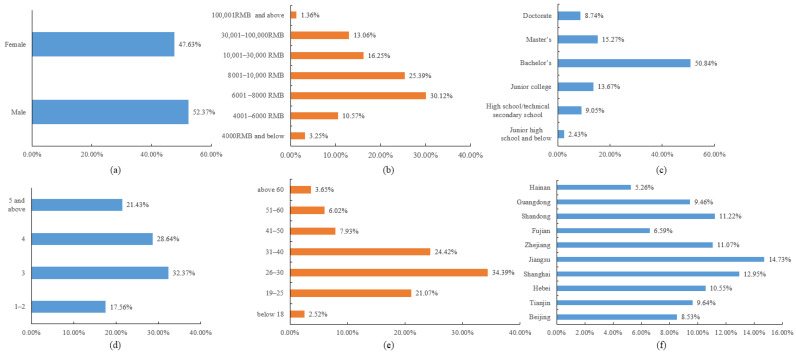
Demographic characteristics of the sample (N = 1732): (**a**) gender, (**b**) monthly income, (**c**) educational level, (**d**) family member, (**e**) age, and (**f**) province.

The demographic distribution of the final sample is shown in Figure 3. Of all samples, 52.37% are male and 47.63% are female. Respondents aged 26 to 40 accounted for 58.81%, and 61.01% of the respondents live in a family of three or four members. In total, 74.85% of the respondents have achieved a bachelor’s degree or above in terms of educational level; 55.51% of the respondents have a monthly income between RMB 6000 and 8000, and nearly half (49.97%) of the sample is from Jiangsu, Shanghai, Zhejiang, and Shandong.

### 4.2. Effects of Individual–Group-Level Factors

Before analyzing the data collected by questionnaires, a normality test was carried out using SPSS 22.0. The results showed that the data is normally distributed, indicating that the data is suitable for regression analysis. With Stata 16 applied, the effects of individual–group-level factors on *PLCBs* are checked according to Equation (1), and the results are included in Table 2.

As shown in Table 2, the F-value of the regression model is 241.231, and it is significant at the 1% level. The R^2^ and the adjusted R^2^ are 0.515 and 0.514, respectively, indicating that the regression model has a goodness of fit. The regression coefficients of *NI*, *LC*, *SC*, *EL*, and *EC* are positive and significant at 1%, suggesting that normative internalization, learning capacity, symbol concern, expertise level, and environmentalism culture are positively associated with *PLCBs*. Thus, H1, H2, H3, H4, and H6 are valid. Moreover, learning capacity and normative internalization on *PLCBs* are more significant than other factors, with regression coefficients of 0.271 and 0.260, respectively. The regression coefficient of *CC* is −0.125 and significant at the 1% level, indicating that the culture of consumerism negatively influences *PLCBs*. Therefore, H5 is supported.

### 4.3. Impact of Psychological Empowerment Perception

According to the three steps shown in Section 3.3.2, the mediating effect of psychological empowerment perception on the association between individual–group-level factors and *PLCBs* is checked. The ratio of the mediating effect to the full impact is also calculated according to Equation (2), as demonstrated in Table 3.

The results showed that all the coefficients are significant in six paths, denoting that psychological empowerment perception mediated the relationships between normative internalization, learning capacity, symbol concern, expertise level, consumerism culture, environmentalism culture, and *PLCBs*. Therefore, H7 is confirmed. Regarding the ratio of the mediating effect to the full impact, the maximum is 62.64% in the path of “*SC*-*PEP*-*PLCBs*”. The minimum is observed in the path of “*LC*-*PEP*-*PLCBs*”, with a proportion of 20.51%.

## 5. Discussion

As spontaneous and voluntary activities, *PLCBs* are crucial to achieving carbon neutrality and the UN’s sustainable development goals. With a survey conducted in eastern China, this study found that the mean value of *PLCBs* is 3.51, suggesting that residents’ responses to *PLCBs* are encouraging. Using the TOPSIS method, Wang et al. [63] calculated the low-carbon development quality of 259 cities in China and found that cities with better quality are mainly located in eastern regions, supporting the results found in this study. Using data collected from 716 participants in Australia, O’Brien et al. [64] measured the low-carbon readiness index and found its mean value and the standard deviation are 3.81 and 0.91, respectively. This is consistent with the findings in this study. It was confirmed that normative internalization and learning capacity positively influence *PLCBs*. Cruwys et al. [65] also found that normative change plays a pivotal role in the behavioral choice and can be used in group interventions. It was found that normative internalization can strengthen individual social responsibility and ecological value and enhance their involvement in pro-environmental behaviors [66]. In terms of learning capacity, adaptive and generic learning was helpful in managing conflicts among stakeholders and achieving collective actions [67]. Moreover, organizational roles, collective interests, and collaborative culture can be fully completed by enhancing individual learning capacity [68]. Therefore, normative internalization and learning capacity provide new ways to promote *PLCBs*.

Descriptive statistics indicated that the mean value of symbol concern is 3.92, and the main effect test showed that symbol concern has a positive effect on *PLCBs*. This implied that the residents are concerned about the symbols embedded in *PLCBs*, and this kind of particular sign is conducive to engaging residents in low-carbon actions. It was found that the symbolic attribute of electric vehicles (i.e., opening, freedom, and environmental protection) can improve consumers’ willingness to pay [43]. Straus [69] stated that the symbols endowed in public policy reflect the individual requirement for identification, expectations, and support for policy frameworks. After proposing four attributes related to low-carbon innovation (public functional, private functional, public symbolic, and private symbolic), Pettifor et al. [70] asserted that added value in the public sphere associated with low-carbon innovation must be outlined, which is a support for the findings in this study. The effects of symbol concern on the *PLCBs* can be explained by self-influence and interpersonal influence. In terms of self-influence, individuals can apply the symbols endowed in *PLCBs* to construct, enhance, display, and distinguish themselves [71]. Regarding interpersonal influence, the symbols embedded in the *PLCBs*, such as health and responsibility, enable individuals to Convery their status, reputation, and group attribute to the outside world [40]. Consequently, the symbol lies in *PLCBs* should be elaborately identified, and practical measures should be launched to enforce individual symbol concerns.

It was found that psychological empowerment perception mediated the association between individual–group-level factors and *PLCBs*. As a perception of being “authorized”, psychological empowerment perception acts as a “conductor”, by which motivations convert into expected behaviors. In other words, residents’ positive psychological tendencies, such as environmental concern, ecological value, and attitude, can induce *PLCBs* through psychological empowerment perception. In this context, individuals are confident in conducting pro-environmental activities and believe they are entitled to protect the environment. Previous studies have confirmed the positive effect of psychological empowerment perception. Using data collected from employees in Canada, Paille and Francoeur [72] discovered that psychological empowerment promoted the required green-task performance. Moreover, only when all components of empowerment (including competence, meaning, self-determination, and impact) are presented can highly required green-task performance be achieved. After surveying public-sector employees, Garcia-Juan et al. [73] discovered that psychological empowerment was highly associated with effective commitment and job satisfaction. In environmental behavior, it was found that psychological empowerment moderated the relationship between an organizational innovative climate and individual innovative behavior [74]; the association between ecological, moral education, and pro-environmental behaviors was partly mediated by psychological empowerment [75]. Consequently, *PLCBs* can be encouraged by enforcing individual psychological empowerment of low-carbon-related activities.

## 6. Conclusions, Recommendations, and Limitations

*PLCBs* are crucial to achieving carbon neutrality, and identifying factors influencing *PLCBs* is indispensable to changing residents’ consumption patterns. In this study, a model was proposed from an individual–group level to determine the influencing factors of *PLCBs*, with some critical findings obtained. First, *PLCBs* are positively influenced by normative internalization, learning capacity, symbol concern, expertise level, and environmentalism culture. In particular, the effects of learning capacity and normative internalization on *PLCBs* are more significant than other factors, with regression coefficients of 0.271 and 0.260, respectively. Second, consumerism culture hurts *PLCBs* with an influence coefficient of −0.125. Finally, psychological empowerment perception partly mediated the association between individual–group-level factors and *PLCBs*, and the maximum ratio of the mediating effect to the full impact is 62.64%.

*PLCBs* are positively influenced by individual–group-level factors, such as normative internalization, learning capacity, symbol concern, and environmentalism culture. Therefore, intervention measures should be made to engage individuals in *PLCBs*. Public-service announcements, community activities, and knowledge competitions should be conducted to improve residents’ learning environment and enhance their involvement in *PLCBs*. The symbols contained in *PLCBs* should be elaborately identified, and propaganda should be implemented to guide the public to identify and pursue the symbols. Professionals can be invited to the community to share experience and knowledge related to low-carbon consumption. Moreover, the effects of psychological empowerment perception on *PLCBs* cannot be neglected. The significance of *PLCBs*, such as the economic and health benefits, should be explained to an attentive public through education and propaganda. More efforts should be made to improve the infrastructure and low-carbon product catalog to increase residents’ confidence in conducting *PLCBs*. Additionally, the influence of *PLCBs* should be outlined to motivate individuals to be involved in low-carbon activities. For instance, well-awarded examples should be set up, thereby encouraging more individuals to take low-carbon actions.

This study has some possible limitations, which should be addressed in the future. First, data should be collected from more regions and countries to obtain more universal conclusions. Second, longitudinal research should be conducted to track the dynamic and causal relations between the individual–group-level factors and *PLCBs*. Finally, only the mediating effect of psychological empowerment perception was considered in the present study; more emphasis should be put on the other mediating variables involved in *PLCBs*, such as psychological distance and moral attentiveness.

## Figures and Tables

**Table 1:** Descriptive statistics of variables.

	PLCBs ^1^	NI ^2^	LC ^3^	SC ^4^	EL ^5^	CC ^6^	EC ^7^	PEP ^8^
Mean	3.51	2.74	3.04	3.92	3.11	3.75	3.72	3.55
Standard deviation	0.742	0.632	0.648	0.736	0.917	1.137	0.599	0.743

^1^ PLCBs: proactive low-carbon behaviors; ^2^ NI: normative internalization; ^3^ LC: learning capacity; ^4^ SC: symbol concern; ^5^ EL: expertise level; ^6^ CC: consumerism culture; ^7^ EC: environmentalism culture; ^8^ PEP: psychological empowerment perception.

**Table 2:** Regression results of individual–group-level factors on the PLCBs.

Variable	Unstandardized Coefficient	Standard Error	T
NI ^1^	0.260 ***	0.032	13.208
LC ^2^	0.271 ***	0.015	14.457
SC ^3^	0.141 ***	0.013	8.715
EL ^4^	0.105 ***	0.027	6.488
CC ^5^	−0.125 ***	0.018	−7.526
EC ^6^	0.151 ***	0.022	3.184
AG ^7^	0.051 ***	0.013	3.128
GE ^8^	0.032 ***	0.019	2.972
IC ^9^	0.043 **	0.020	2.545
ED ^10^	−0.006	0.052	−0.545
FS ^11^	−0.038 ***	0.007	−3.214
R ^2^	0.515		
Adjusted R ^2^	0.514		
F	241.231 ***		

^1^ NI: normative internalization; ^2^ LC: learning capacity; ^3^ SC: symbol concern; ^4^ EL: expertise level; ^5^ CC: consumerism culture; ^6^ EC: environmentalism culture; ^7^ AG: age; ^8^ GE: gender; ^9^ IC: income; ^10^ ED: educational level; ^11^ FS: family size. *** and ** represent *p* < 1% and *p* < 5%, respectively.

**Table 3:** Mediating effects of psychological empowerment perception.

Path	c_1_	a	b	c_2_	Is There a Mediating Effect?	Ratio of Mediating Effect
NI ^1^-PEP ^2^-PLCBs ^3^	0.260 ***	0.184 ***	0.341 ***	0.198 ***	Yes	24.13%
LC ^4^-PEP-PLCBs	0.271 ***	0.163 ***	0.341 ***	0.213 ***	Yes	20.51%
SC ^5^-PEP-PLCBs	0.141 **	0.259 ***	0.341 ***	0.055 ***	Yes	62.64%
EL ^6^-PEP-PLCBs	0.105 ***	0.178 ***	0.341 ***	0.046 *	Yes	57.81%
CC ^7^-PEP-PLCBs	−0.125 ***	−0.159 ***	0.341 ***	−0.070 ***	Yes	43.38%
EC ^8^-PEP-PLCBs	0.151 **	0.103 ***	0.341 ***	0.112 ***	Yes	23.26%

^1^ NI: normative internalization; ^2^ PEP: psychological empowerment perception; ^3^ PLCBs: proactive low-carbon behaviors; ^4^ LC: learning capacity; ^5^ SC: symbol concern; ^6^ EL: expertise level; ^7^ CC: consumerism culture; ^8^ EC: environmentalism culture. ***, ** and * represent *p* < 1% *p* < 5%, and *p* < 10%, respectively.

## Data Availability

Not applicable.

## Supplementary Materials

The following supporting information can be downloaded at https://www.mdpi.com/article/10.3390/ijerph19106307/s1: Table S1: Measurement items of variables, reliability, and validity analysis.
