# Supplementary material for: Determinants of Proactive Low-Carbon Consumption Behaviors: Insights from Urban Residents in Eastern China
Source: Int J Environ Res Public Health. 2022 May 23;19(10):6307. doi: 10.3390/ijerph19106307 (PMC9141223; doi:10.3390/ijerph19106307)
Supplement: Supplementary file 1 [file ijerph-19-06307-s001.zip › ijerph-1703022-supplementary.pdf]

Table S1. Measurement items of variables, reliability, and validity analysis.

Measurement Items	Cronbach's α	KMO	Factor Loading	CR	AVE
**Proactive Low-carbon Consumption Behaviors (PLCBs)**					
I am used to turning off the lights when leaving the room.					
It is my habit to bring shopping bags when going to the supermarket.					
I have developed the habit of checking carbon labels when buying products.					
I give priority to purchasing energy-saving home appliances because they can save money.					
I prioritize using renewable and recycled items because they are suitable for the environment.					
I prioritize riding when going out because it is beneficial to my health.	0.765	0.773	0.812	0.774	0.536
I always encourage my family to save energy.					
I always encourage my colleagues to buy new energy vehicles instead of fuel vehicles.					
I buy energy-saving appliances because the people around me buy them.					
I actively participate in low-carbon-related actions held by the community.					
I provide advice for the government to save electricity and water.					
I will report to the community when I observe some wasting water in the living district.					
**Normative internalization (NI)**					
Saving water and electricity is what I should do well.					
I will save energy even if there is no supervision.	0.843	0.851	0.872	0.757	0.522
I will feel guilty if I buy energy-consuming appliances.					
**Learning capacity (LC)**					
I refer to experience when buying home appliances.					
I broaden my knowledge of low-carbon consumption in various ways.	0.876	0.817	0.807	0.757	0.515
I can correct my understanding of low-carbon consumption.					
**Symbol concern (SC)**					
I can express my personality by practicing low-carbon consumption behaviors.	0.786	0.802	0.834	0.792	0.563

Measurement Items	Cronbach's α	KMO	Factor Loading	CR	AVE
Practicing low-carbon consumption behaviors improve others' impression of me.					
Practicing low-carbon consumption behaviors demonstrates social status.					
Practicing low-carbon consumption behaviors enable me to gain a higher social reputation.					
**Expertise level (EL)**					
People around me know a lot about the low-carbon consumption guiding policy.					
Professionals with low-carbon-related knowledge surround me.	0.845	0.756	0.821	0.787	0.518
People around me have unique views on low-carbon-related policies and behaviors.					
**Consumerism culture (CC)**					
It is widespread that people consume goods and services in large quantities.					
It is widespread that people consume luxury goods and services.	0.837	0.826	0.789	0.802	0.521
It is widespread to observe over-consumption.					
**Environmentalism culture (EC)**					
Many people pay attention to emissions reduction and energy saving.					
A lot of people attach great importance to conserving energy.	0.842	0.805	0.806	0.788	0.547
Many people have made efforts to change their behaviors to protect the environment.					
**Psychological empowerment perception (PEP)**					
I think it makes sense to practice low-carbon consumption behaviors.					
I think it is pivotal to practice low-carbon consumption behaviors.					
I think it is necessary to practice low-carbon consumption behaviors.					
I can well practice low-carbon consumption behaviors.					
I have the confidence to practice low-carbon consumption behaviors.	0.794	0.803	0.779	0.792	0.564
It would be helpful to persuade others to practice low-carbon consumption behaviors.					
I have autonomy in how to practice low-carbon consumption behaviors.					
I can decide how to persuade others to practice low-carbon consumption behaviors.					
I have the right to decide whether to report the people violating low-carbon-related policy.					

Measurement Items	Cronbach's α	KMO	Factor Loading	CR	AVE
Practicing low-carbon consumption behaviors can improve the living environment.					
I can persuade people around me to practice low-carbon consumption behaviors.					
My suggestions on low-carbon consumption behaviors can get the response of the government.					
